# Coverage models to determine outreach vaccination center locations in low and middle income countries

**DOI:** 10.1016/j.orhc.2016.02.003

**Published:** 2016-06

**Authors:** Jung Lim, Erin Claypool, Bryan A. Norman, Jayant Rajgopal

**Affiliations:** Department of Industrial Engineering, University of Pittsburgh, 1025 Benedum Hall, Pittsburgh, PA 15261, United States

**Keywords:** Vaccine delivery, Outreach, Planning, Location, Covering

## Abstract

The Expanded Programme on Immunization (EPI) was established in 1974 to ensure that children all around the world benefit from life-saving vaccines. However, in many low and middle income countries, it is extremely difficult to vaccinate the entire population with the standard regimen of vaccines. One important reason for this is geographically dispersed or nomadic populations. To improve vaccination rates, these countries typically use outreach, where health workers take vaccines to remote locations. Outreach is the last, critical link in the vaccine supply chain, and the locations selected to offer outreach directly impact the number of additional children that can be vaccinated. This research presents four quantitative models that can be used to optimize the selection of outreach locations, in order to maximize the number of residents that can be reached; each model addresses a different type of coverage possibility. The models are analyzed and contrasted using an example with inputs generated from a subset of data from the state of Bihar in India that was made available to the authors.

## 1 Introduction

The Expanded Programme on Immunization (EPI) was established in 1974 by the World Health Organization (WHO) to ensure that children all around the world benefit from life-saving vaccines  [1]. However, vaccine delivery in many low and middle income countries is an extremely complex problem. The supply chains in such countries are limited in their cold-storage capacity and in their ability to transport vaccines quickly to various points throughout the country. In addition to these supply chain limitations, many of these countries have geographically dispersed or nomadic populations. Portions of their populations have limited or no access to vaccination locations due to poor infrastructure (poor road conditions or limited transportation) or other geographic barriers. As examples, in the country of Niger, 90% of the roads are unpaved  [2]. In Nigeria, people from some rural areas may have to walk at least 26 miles to access health care  [3]. In Kenya, 40% of the population must travel in excess of an hour to the nearest primary healthcare facility  [4]. Thus, people from remote locations within resource-deprived countries have difficulty reaching immunization locations for their standard regimen of vaccines. This puts these individuals at a very high risk of mortality from infectious diseases such as measles, yellow fever, polio and tuberculosis.

One method to overcome this challenge is to use *outreach*. Sustained outreach is a strategy for reaching remote sections of the population with limited access to immunization locations. With this service, health care workers take vaccines from a fixed immunization location and travel to the remote locations, to immunize individuals there. This service is different from a *campaign* which is a one-time attempt to raise immunization rates. Outreach is extremely important to the overall immunization programs in resource-deprived countries. Without outreach, many countries would suffer from extremely low coverage rates. For example, a study was carried out in three zones of different population densities within Kenya to test the effectiveness of outreach programs as compared to only utilizing fixed immunization locations. The study showed that, with outreach, the coverage rate increased from 25% to 57% in the zone with lowest population density. Coverage increased from 54% to 82% in the zone with greatest population density  [5].

Outreach is typically provided on a systematic basis, at regular time intervals and regular outreach locations. However, the outreach activities conducted from each immunization location can vary greatly depending on financial resources, time constraints, vaccine availability, population characteristics, usage rate of the fixed immunization location, health worker training, portable cold chain equipment available, and transportation available. The decisions about when and where to conduct outreach and which vaccines to administer may be made locally, depending on each location’s available resources  [6].

Outreach from health centers constitutes the critical final link in the vaccine supply chain, which can be quite complex and is typically comprised of four levels in addition to outreach: a central location where vaccines are received into the country from manufacturers, regional locations (typically five to 10) that serve as distribution hubs, districts (typically 25–100) which serve as the next layer of distribution and where some vaccinations may occur, and immunization health centers (typically 100–2000) which provide vaccinations to patients  [7]. Outreach planning has a significant effect on the behavior of the entire vaccine distribution chain. As previously noted, in many countries successful outreach greatly increases the number of people vaccinated and therefore increases the number of vaccines that must flow through the entire vaccine supply chain. Thus, it is vital that countries consider the design and intended operation of their outreach programs as they are designing and equipping their entire vaccine supply chain.

In summary, vaccine delivery is a complicated problem and the effectiveness of delivery is critical to reducing mortality rates in many resource-deprived countries. To increase effectiveness, outreach is widely utilized. However, there are no quantitative outreach planning models available to help countries and individual facilities plan the optimal outreach strategy. The purpose of this research is to address this need.

## 2 Problem development and literature review

The objective in each of the various models formulated in this paper is to maximize the number of people vaccinated through outreach, when resources are limited. We assume that outreach is necessary whenever one or more villages are more than a distance $D_{1}$ (typically, 5 km as per WHO guidelines  [8]) from an existing Immunization Health Center (IHC). An outreach team from the IHC visits one or more such villages, and residents from that village and all villages that are within a distance $D_{1}$ of it are able to go there to be vaccinated. We refer to a village that serves as an outreach vaccination center as a “center” and the other nearby villages (within distance $D_{1}$) from which residents travel to the center as “satellite” villages. The maximum number of centers that can be selected for outreach during the planning horizon depends on the financial and other resources available at the IHC. The objective is to select centers so as to maximize the number of residents that can be served at each of the central villages and its respective satellite villages.

**Fig. 1:**
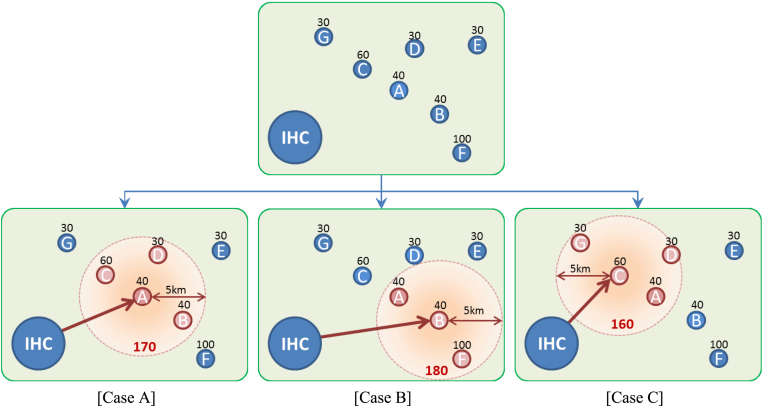
Outreach example: selecting an outreach location.

As an illustrative example, Fig. 1 shows seven villages (represented by the small circles) located near an IHC along with their corresponding patient populations (represented by the numbers above the circles). Three options are shown for the selection of an outreach center from that IHC. If village A is selected as the center [Case A], then the satellite villages that are within 5 km are villages B, C, and D, and thus people in villages A, B, C and D can be vaccinated. People in villages E, F and G will not be vaccinated. In this case, the number of residents that can be covered by outreach is 170. Similarly, 180 people can be covered in Case B and 160 in Case C. Therefore, if we are restricted to a single outreach location, then among these three villages, B would be the best option for a center.

While more than one outreach strategy might be possible, there will typically be constraints that limit the final choice of outreach options. For example, outreach to a particular location has a cost associated with it (that might depend upon distance or terrain or equipment used) and there might be some overall budget for outreach that constrains our choice of outreach trips. Alternatively, costs might be similar for outreach to different sites but we might have a direct limit on the number of outreach sessions (e.g., because of personnel, vehicle, or equipment limits). In other cases, there might be limits on the length of a trip or preferences for certain trips over others. Different strategies are possible depending on these constraints and the assumptions made on the type and amount of patient coverage that can be obtained at a center.

Prior research that most closely relates to that described in this work is reported in a paper by Verter and Lapierre  [9], who address the location of preventive health care facilities to maximize participation, under the assumption of a linear decrease in participation probabilities as distance to the nearest facility increases. They present an integer programming formulation and illustrate results using data from two locations in the US and Canada. These authors as well as Daskin and Dean  [10] discuss how the location set covering model, maximal covering model and P-median model have been used for location planning in health care and reviewed other models derived from these three basic facility models. The different model types are applied selectively according to a problem’s characteristics and objective. The problem addressed here may be viewed as a covering problem, which is well-known among facility location models  [11]. In particular, it is related to the Maximal Covering Location Problem (MCLP), which was developed by Church and ReVelle  [12], with the objective of maximizing the amount of demand covered by a facility. In this model, it is assumed that all of the demand is covered if the demand location is within an acceptable service distance, otherwise it is not, i.e., coverage is binary. An extension to this is the concept of partial coverage, in which there are two distances: the maximum full coverage distance $D_{1}$ and the minimum non-coverage distance $D_{2}$. The demand within distance $D_{1}$ from a facility is fully covered while none of the demand beyond distance $D_{2}$ is covered. For demand at locations between distances $D_{1}$ and $D_{2}$ from the facility, the coverage level is assumed to be a decreasing function of the distance to the demand location. Thus, some customers are fully covered and the others are partially covered  [13]. This variation has been called the gradual covering problem by Drezner, Wesolowsky, and Drezner  [14], or MCLP with partial coverage by Karasakal and Karasakal  [15]. Berman and Krass  [13] collectively refer to this class of models as the Generalized Maximal Covering Location Problem (GMCLP). In order to apply linear programming, they assume that the decreasing function for partial coverage is stepwise, so that the model is similar to MCLP. In these models, all of the demand at a location is assigned to the nearest facility, even though there might be two or more facilities near the demand location that are capable of serving the demand. Berman, Drezner, and Krass  [16] introduce the cooperative coverage model where the effect of facilities is combined if there are more than two facilities near the demand location. However, in this model the coverage is once again binary, with a demand location being fully covered if an aggregation of partial coverage possible from nearby facilities exceeds a certain threshold; otherwise there is no coverage. That is, there is no partial coverage of demand points.

## 3 Coverage models

In this paper, we consider four types of models to optimize coverage from outreach. In all of our models we consider multiple outreach locations that can be selected. We start with a basic model that is similar to the binary MCLP model. The second model extends this by drawing from the GMCLP approach, with coverage being a stepwise and decreasing function of distance. The third model is a new generalization of the cooperative cover model: rather than being binary, an accumulation of partial coverage becomes the partial coverage of the location. The final model is a larger one that could be viewed as a generalization of any of the first three models. Here we formulate it as an extension of the second one, where each center is constrained to lie within a given radial distance from one of several specific points (the IHCs). For ease of exposition, we assume that there is sufficient capacity to vaccinate the people who are targeted by an outreach trip (although it would be a straightforward extension to add in capacity constraints for trips). These models are described in the next four subsections, followed by numerical illustrations of each in the section after that. The illustrations use data that is generated from partial information on the state of Bihar in northern India that was made available to the authors, and which was the motivating application for this work. We conclude with a discussion and summary of our work in the final section.

### 3.1 Model 1: Binary coverage model

In this basic model, it is assumed that residents in villages within a radius of $D_{1}\;\mathrm{km}$ from an outreach center are covered, while residents in other villages are not.

*Notation*:

n: Total number of villages to be served via outreach from the IHC
$p_{i}$: Number of residents living in village i
$c_{i}$: Cost of outreach at village i if it serves as an outreach center
$d_{ij}$: Distance between village i and village j (with $d_{ii}=0$)
$D_{1}$: Maximal coverage distance
C: Available budget for outreach
N: Maximum number of outreach centers that is feasible
$x_{i}\in \left\{0,1\right\}$; 1 if village i is selected as an outreach center; 0 otherwise
$y_{i}\in \left\{0,1\right\}$; 1 if village i is covered; 0 otherwise.

The mathematical model is as follows:

(1) $$\mathrm{Max}\overset{n}{\underset{i=1}{\sum }}p_{i}y_{i}$$

s.t.

(2) $$y_{i}\leq \underset{j\in S_{i}}{\sum }x_{j}\;\text{for }S_{i}=\left\{j:d_{ij}\leq D_{1},\;j=1,\ldots ,n\right\},i=1,\ldots ,n$$

(3) $$\overset{n}{\underset{i=1}{\sum }}{c_{i}x}_{i}\leq C$$

(4) $$\overset{n}{\underset{i=1}{\sum }}x_{i}\leq N$$

(5) $$x_{i}\in \left\{0,1\right\}\text{,}\;y_{i}\in \left\{0,1\right\}\text{,}\;\text{for }i=1,\ldots ,n\text{.}$$

The objective is to maximize the number of people who are vaccinated by outreach (across all villages selected along with their respective satellites). Constraint (2) ensures that village i is covered only if it is $D_{1}$ km or less from any village j which serves as an outreach center (a typical value for $D_{1}$ might be 5 km). Constraints (3), (4) respectively ensure that the available outreach budget and the limit on the number of outreach centers are not exceeded. It is conceivable that only one of these constraints might exist.

### 3.2 Model 2: variable single coverage model

In this model, it is assumed that the coverage by outreach is a stepwise decreasing function of distance from an outreach center, rather than being binary. Given $D_{1}<D_{2}<\cdots <D_{K}$ and $1=\alpha_{1}>\alpha_{2}>\cdots >\alpha_{K}>0$, coverage is divided into groups:

• If there are centers within distance $D_{1}$ of the village, all residents (i.e., a fraction $\alpha_{1}=1$) go to one such center; else
• If there are centers between distance $D_{1}$ and $D_{2}$, then a fraction $\alpha_{2}$ of the patient population will choose to visit one such center; else
• …
• If there are centers between distance $D_{K-1}$ and $D_{K}$, then a fraction $\alpha_{K}$ of the population will choose to visit one such center; else
• There is no coverage.

A typical example might be K=3 with $D_{1}=5\;\mathrm{km}$, $D_{2}=8\;\mathrm{km}$, $D_{3}=10\;\mathrm{km}$, and $\alpha_{1}=1$, $\alpha_{2}=0.5$, $\alpha_{3}=0.2$.

Additional notation is as follows:

$D_{k}$: Distance from the outreach center of the kth coverage boundary, k=1,2,…,K
$\alpha_{k}$: Coverage fraction attained if the nearest center is between $D_{k-1}$ and $D_{k}\;\mathrm{km}$ of a village.

Instead of the $y_{i}$ variables of the prior section we now have

$y_{ik}\in \left\{0,1\right\}$; 1 if village i is covered by a center between $D_{k-1}$ and $D_{k}\;\mathrm{km}$ of it;
0 otherwise.

The model is as follows:

(6) $$\mathrm{Max}\overset{n}{\underset{i=1}{\sum }}p_{i}\overset{K}{\underset{k=1}{\sum }}{\alpha_{k}y}_{ik}$$

s.t.

(7) $$y_{ik}\leq \underset{j\in S_{i}}{\sum }x_{j}\;\text{for}S_{i}=\left\{j:{D_{k-1}\leq d}_{ij}\leq D_{k},j=1,\ldots ,n\right\},\;i=1,\ldots ,n\text{;}$$

k=1,…,K

(8) $$\overset{K}{\underset{k=1}{\sum }}y_{ik}\leq 1\;\text{for }i=1,\ldots ,n$$

(9) $$\overset{n}{\underset{i=1}{\sum }}{c_{i}x}_{i}\leq C$$

(10) $$\overset{n}{\underset{i=1}{\sum }}x_{i}\leq N$$

(11) $$x_{i}\in \left\{0,1\right\}\text{,}\;y_{ik}\in \left\{0,1\right\}\text{,}\;\text{for }i=1,\ldots ,n,\;k=1,\ldots ,K\text{.}$$

In this model the objective is the same as in the previous model but coverage is according to the appropriate coverage fraction. Constraint (7) ensures that $y_{ik}$ can be 1 only if village i is within the appropriate coverage radius from any outreach center. Constraint (8) ensures that village i is assigned to at most one outreach center. Constraints (9), (10) are the usual budget/resource constraints akin to (3), (4) in Model 1.

### 3.3 Model 3: variable multiple coverage model

This model is a generalization of the previous model: villages that are not within the 100% coverage distance $D_{1}$ are not restricted to partial coverage by a single center (unless it is the only available choice). Rather, residents who do not visit one such center might choose to visit another one. More specifically, given $D_{1}<D_{2}<\cdots <D_{K}$ and $1=\alpha_{1}>\alpha_{2}>\cdots >\alpha_{K}>0$, coverage follows the following pattern:

• If there are $m_{1}>0$ centers within distance $D_{1}$ of the village, all residents (i.e., a fraction $\alpha_{1}=1$) go to one such center; else
• If there are $m_{2}>0$ centers between distance $D_{1}$ and $D_{2}$, then a fraction $\alpha_{2}$ of the population will choose to visit one such center; a further fraction $\alpha_{2}$ of the remaining population will choose to visit another such center; and so on
• …
• If there are $m_{K}>0$ centers between distance $D_{K-1}$ and $D_{K}$, then a fraction $\alpha_{K}$ of the remaining population will choose to visit one such center; a further fraction $\alpha_{K}$ of the remaining population will choose to visit another such center; and so on
• There is no coverage if there is no center within distance $D_{K}$ of the village.

In general, the coverage in a village would be given by

(12) $$\beta =1-\overset{K}{\underset{k=2}{\prod }}{\left(1-\alpha_{k}\right)}^{m_{k}}\text{.}$$

**Fig. 2:**
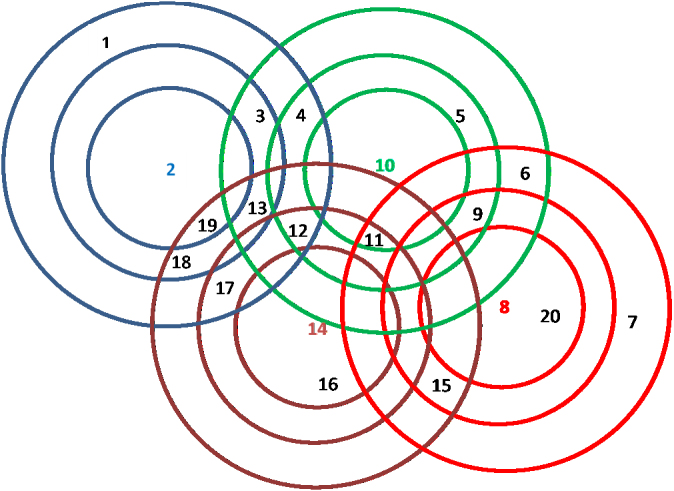
Variable outreach coverage example.

As an example, with K=3, $D_{1}=5\;\mathrm{km}$, $D_{2}=8\;\mathrm{km}$, $D_{3}=10\;\mathrm{km}$, $\alpha_{1}=1$, $\alpha_{2}=0.5$, $\alpha_{3}=0.2$, $m_{1}=0$, $m_{2}=2$, $m_{3}=1$, the fraction of residents covered would be given by $1-{\left(1-0.5\right)}^{2}{\left(1-0.2\right)}^{1}=0.80$. Thus, if the village had 100 residents, since there are no centers in the inner circle, 50% (i.e., 50) would go to one of the two centers in the next circle while 50% of the remaining 50 (i.e., 25) would go to the other, and 20% of the remaining 25 (i.e., 5) would go to the center in the outer circle; 20 residents would choose not to go to any center for immunization. To further illustrate the difference between the model in this section and the previous one, consider Fig. 2 with four outreach centers in a region of 20 villages; these centers are located at villages 2, 8, 10 and 14. Suppose that as before $\alpha_{1}=1$, $\alpha_{2}=0.5$ and $\alpha_{3}=0.2$ in both models.

Consider village 6 and 11 neither of which is within the inner circle of any center and thus cannot receive 100% coverage. Village 6 is within the outer circles of centers located at villages 8 and 10: with Model 2, the coverage would be 20%, all at one of centers 8 or 10. With Model 3, the coverage would be 36%: 20% at one of 8 or 10, and 16% (i.e., 20% of the remaining 80%) at the other. Village 11 is within the middle circle of the centers at locations 10 and 14 and within the outer circle of the center at location 8. Here the coverage would be 50% with the first model (at either center 10 or center 14), but in the second model with three possible center options, it would be $1-{\left(1-0.5\right)}^{2}\left(1-0.2\right)=80\%$ (50% at one of villages 10 or 14 and 25% at the other, 5% at village 8).

In our formulation of this problem we restrict ourselves to K=3. Define the following additional notation:

$M_{r}$: Maximum number of villages within the rth coverage circle of any village, r=1,2,3;
$\beta_{m_{2}m_{3}}$: Coverage constant with $m_{2}$ centers between $\left(D_{1},D_{2}\right)$;
  $$m_{3}\text{ centers between }\left(D_{2},D_{3}\right)=1-{\left(1-\alpha_{2}\right)}^{m_{2}}{\left(1-\alpha_{3}\right)}^{m_{3}}$$
$z_{ikl}\in \left\{0,\;1\right\}$;
1 if there are no centers located within distance $D_{1}$ of i, k centers located between distance $\left(D_{1},D_{2}\right)$ of i, and l centers located between distance $\left(D_{2},D_{3}\right)$ of village i; 0 otherwise.
Instead of the $y_{ik}$ variables of the prior section we now have
$y_{i}\in \left\{0,\;1\right\}$;
1 if there is at least one center located within distance $D_{1}$ of i; 0 otherwise.

The values of $M_{r}$ are determined *a priori* by preprocessing. To illustrate the notation, consider the outreach assignment shown in Fig. 2. For village 11, we have 0 centers within distance $D_{1}$, 2 centers between distance ($D_{1}$, $D_{2}$), and 1 center between distance ($D_{2}$, $D_{3}$). Thus $z_{11,2,1}=1$ and $z_{11,k,l}=0$ for all other k, l. For village 19, the corresponding numbers are 1, 0, and 2, but the model will insure $z_{19,k,l}=0$ for all k, l because there is a center (at location 2) within distance $D_{1}$ of village 19.

The model is as follows:

(13) $$\mathrm{Max}\overset{n}{\underset{i=1}{\sum }}p_{i}y_{i}+\overset{n}{\underset{i=1}{\sum }}\overset{M_{2}}{\underset{k=0}{\sum }}\overset{M_{3}}{\underset{l=0}{\sum }}p_{i}\beta_{kl}z_{ikl}$$

s.t.

(14) $$y_{i}\leq \underset{j\in S_{i}}{\sum }x_{j}\;\text{for }S_{i}=\left\{j:d_{ij}\leq D_{1},\;j=1,\ldots ,n\right\},\;i=1,\ldots ,n$$

(15) $$\overset{M_{2}}{\underset{k=0}{\sum }}\overset{M_{3}}{\underset{l=0}{\sum }}z_{ikl}+y_{i}\leq 1$$

(16) $$\overset{M_{2}}{\underset{k=0}{\sum }}k\overset{M_{3}}{\underset{l=0}{\sum }}z_{ikl}\leq \underset{j\in S_{i}}{\sum }x_{j}\;\text{for}S_{i}=\left\{j:{D_{1}<d}_{ij}\leq D_{2},\;j=1,\ldots ,n\right\},\;i=1,\ldots ,n$$

(17) $$\overset{M_{3}}{\underset{l=0}{\sum }}l\overset{M_{2}}{\underset{k=0}{\sum }}z_{ikl}\leq \underset{j\in S_{i}}{\sum }x_{j}\;\text{for}S_{i}=\left\{j:{D_{2}<d}_{ij}\leq D_{3},\;j=1,\ldots ,n\right\},\;i=1,\ldots ,n$$

(18) $$\overset{n}{\underset{i=1}{\sum }}{c_{i}x}_{i}\leq C$$

(19) $$\overset{n}{\underset{i=1}{\sum }}x_{i}\leq N$$

(20) $$x_{i}\in \left\{0,1\right\}\text{,}\;y_{i}\in \left\{0,1\right\}\text{,}\;\text{for }i=1,\ldots ,n$$

(21) $$z_{ikl}\in \left\{0,1\right\}\text{,}\;\text{for }i=1,\ldots ,n,\;k=0,\ldots ,M_{2},\;l=0,\ldots ,M_{3}\text{.}$$

The objective in this model has two terms: the first one counts the number of residents in villages with 100% coverage and the second in villages that obtain partial coverage. Constraints (14), (18) and (19) are similar to the ones in the prior models, while (15) ensures that if village i gets coverage, it is either 100% coverage or partial coverage from one particular combination of villages in the inner and outer secondary coverage circles. Constraints (16), (17) along with the fact that $\beta_{kl}$ is monotone increasing in k and l ensure that $z_{ikl}=1$ when there are k centers located between distance ($D_{1}$, $D_{2}$) and l centers located between distance ($D_{2}$, $D_{3}$) of village i.

### 3.4 Model 4: model with multiple IHCs

In the final model we consider an entire district with multiple IHCs located within it. It is possible that a particular village might be a candidate for outreach from more than one IHC. This model addresses the problem of developing the best combination of outreach programs across all IHCs within a district. We could embed any of the models of the previous section into a larger problem for the entire district as appropriate; here we illustrate the model using the case where there is variable single coverage at each village (as in Model 2). Additional notation is as follows:

m: Number of different IHCs in the district
$N_{q}$: Maximum number of outreach activities from IHC q
$D_{\max}$: Maximum travel distance to an outreach location from any IHC.
We define $y_{ik}$ in a similar manner to the variable single coverage model but also define
$x_{li}=\left\{0,1\right\}$;1 if village i is selected as a center for outreach from IHC l; 0 otherwise.

The model is as follows:

(22) $$\mathrm{Max}\overset{n}{\underset{i=1}{\sum }}p_{i}\overset{K}{\underset{k=1}{\sum }}{\alpha_{k}y}_{ik}$$

s.t.

(23) $$y_{ik}\leq \underset{j\in S_{i}}{\sum }\overset{m}{\underset{q=1}{\sum }}x_{lj}\;\text{for }S_{i}=\left\{j:{D_{k-1}\leq d}_{ij}\leq D_{k},\;j=1,\ldots ,n\right\},i=1\text{ to }n,\;k=1,\ldots ,K$$

(24) $$\overset{K}{\underset{k=1}{\sum }}y_{ik}\leq 1\;\text{for }i=1,\ldots ,n$$

(25) $$\overset{n}{\underset{i=1}{\sum }}{c_{i}x}_{i}\leq C$$

(26) $$\overset{n}{\underset{i=1}{\sum }}x_{qi}\leq N_{q}\;\text{for }q=1,\ldots ,m$$

(27) $${d_{qi}x}_{qi}\leq D_{\max}\;\text{for }q=1,\ldots ,m,\;j=1,\ldots ,n$$

(28) $$\overset{m}{\underset{q=1}{\sum }}x_{qi}\leq 1\;\text{for }i=1,\ldots ,n$$

(29) $$x_{qi}\in \left\{0,1\right\}\text{,}\;y_{ik}\in \left\{0,1\right\}\text{,}\;\text{for }i=1,\ldots ,n,\;k=1,\ldots ,K;\;q=1,\ldots ,m\text{.}$$

Here C represents the budget for the entire district in (25), a separate limit on the number of outreach sessions is defined for each IHC along with a distance constraint for each IHC in (26), (27), and (28) that ensures that if there is an outreach center at a village it must come from a unique IHC.

## 4 Numerical illustration

We first illustrate the binary, variable single and variable multiple coverage models with the following example based on the Tetia Bambar IHC in the state of Bihar, India. This IHC has a total of 78 villages in its catchment area that are candidates for outreach centers. We were provided with the distances from the IHC to each outreach village and the estimated population of children targeted for vaccination at each village. However, the exact locations of these villages in relation to Tetia Bambar were not available, and given their small sizes and inconsistencies in how their names were spelled it was impossible to accurately locate them on any map. We therefore located the IHC at (0,0) and randomly assigned coordinates to the villages while maintaining the given distances. The resulting coordinates of the villages along with their patient populations are listed in Table 1.

We use coordinate units of 1 km and assume that all distances $d_{ij}$ are Euclidean. For the binary coverage model we assume $D_{1}=5\;\mathrm{km}$. For the variable coverage models, we also assume $D_{2}=8\;\mathrm{km}$ and $D_{3}=10\;\mathrm{km}$ along with coverage fractions $\alpha_{2}=0.5$ and $\alpha_{3}=0.2$. In order to compare the results across the various models we ignored the budget constraints (i.e.,  (3), (9), and (18)) because it was impossible to obtain even approximate estimates from Bihar. We only used the constraints on the maximum number of outreach activities (centers), N (i.e.,  (4), (10), and (19)). We solved each model for increasing values of N until we obtained 100% coverage. For each model, Table 2 lists the coverage obtained for each value of N, along with the respective locations of the outreach centers. The numbers in bold face represent new locations of outreach centers that are added to or replace the ones from the previous (lower) value of N. A standard solver, IBM ILOG CPLEX Optimization Studio 12.6, was used directly to solve these MIP models.

The three models often give different locations and levels of coverage when the limit on the number of centers does not allow for 100% coverage. As an example when only 4 centers are possible the coverage is 80.9% with Model 1, 88.6% with Model 2 and 90.5% with Model 3, and the models do not select the same 4 locations. However, as the number of possible centers (and the corresponding coverage) increases the centers start to converge to the same locations. In all cases, a total of 9 centers are required before 100% coverage can be obtained; the locations are identical and such that each village is within the inner circle (5 km radius) of at least one center. Another interesting observation is that while one new center is always added as we increase N, there are many instances with all models where in addition to adding a new center an existing location is replaced with a new one. This emphasizes the value of an optimization model in selecting the best strategy. As an example, with Models 1 and 2, when N changes from 7 to 8 four of the existing centers are replaced with five new ones; there are only three in common. Similarly, with Models 2 and 3, when N changes from 2 to 3, the two existing locations are replaced by three completely new ones.

Finally, it is worth noting that there could be differences in the actual number of people covered at a specific outreach center; some centers that cover more locations might cater to a larger number of patients than others. However, the imbalances are not substantial. As an illustration, consider the case when we have 6 outreach centers, in which case all three coverage models choose the same set of six locations for outreach as shown in Table 2 (Villages 8, 17, 31, 48, 60 and 73). Table 3 displays the actual population covered at each of these locations under the different coverage models.

**Fig. 3:**
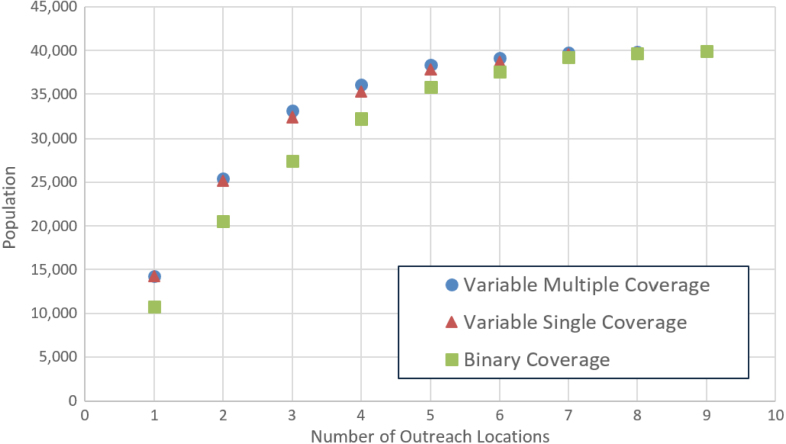
Coverage with first three models.

Fig. 3 provides a visual summary of the coverage results. Obviously, the variable coverage models always provide higher coverage than the binary coverage model but the differences start to get smaller when the number of centers (N) reaches about 7, and the models are identical when N=9. The two variable coverage models behave similarly, and the gains from multiple coverage (as well as from variable coverage) over binary coverage are more noticeable at intermediate values of N. This is significant because in practice, the values of N are more likely to be in this intermediate range: if N is small the options are limited and the benefits of an optimization model are not significant, while large N values are unlikely in practice because of budgetary considerations and resource constraints. While Fig. 3 indicates that we have diminishing marginal gains in coverage as we add outreach sessions, it also allows a social planner to evaluate these gains in light of the extra resources (monetary, equipment, personnel, etc.) that might be required for additional outreach sessions.

**Fig. 4:**
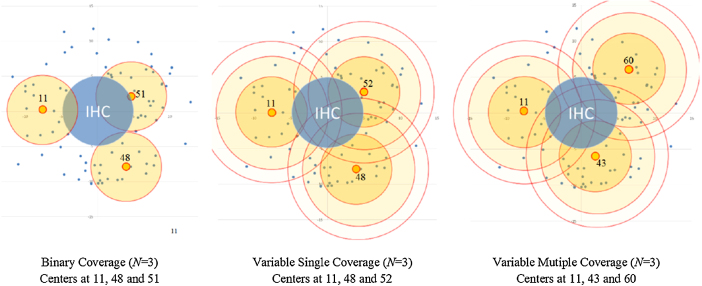
Locations of 6 centers with different types of coverage.

Fig. 4 further illustrates the differences in results from the three models for an intermediate value of N=3. The three panels in the figure provide a visual depiction of the actual locations selected by the models. Notice that location 11 is common to all three models but the others differ depending on the model in use.

**Fig. 5:**
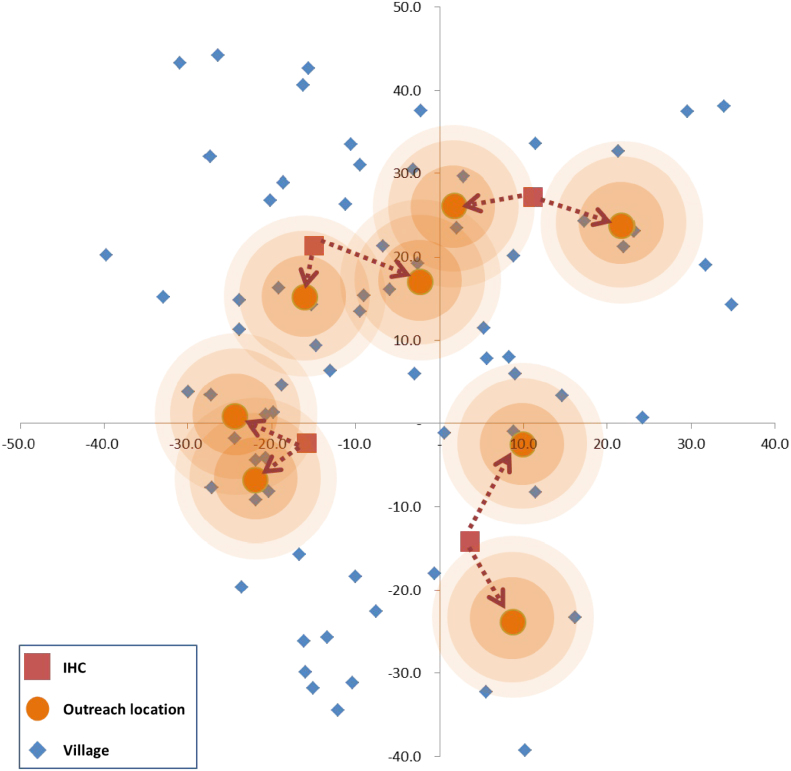
Locations of 6 outreach centers for maximizing coverage.

Finally, to illustrate the multiple IHC model consider a hypothetical district with a total of 80 villages served by 4 IHCs. The locations of the villages and the IHCs are depicted in Fig. 5. Populations of the individual villages are not shown, but these were randomly generated; the total population of the district for this example was equal to 4645.

In defining constraint (26) we assume the same value of $N_{q}$ for all values of q, i.e., that each IHC was restricted to the same maximum number of outreach centers. The multi-IHC problem was solved for values of $N_{q}$ ranging from 1 through 9; the results on the total coverage are shown in Table 4. Once again budget constraints were ignored for the illustration.

As Table 4 indicates, there is a diminishing marginal benefit from allowing an IHC to have an extra outreach center. In practice the number of outreach centers permissible would be limited by the budget and other available resources, but a table such as this one allows planners to balance the additional resources expended with more outreach centers against the gains in the number of residents vaccinated. Fig. 5 illustrates the case where $N_{q}=2$ and shows the locations of the two outreach centers for each of the four IHCs; the total coverage here is about 48%.

## 5 Discussion and summary

To our knowledge the work reported here is the first to provide a formal modeling framework for decision making with respect to outreach. As with any model-based approach, our work has some limitations and certain facts are worth keeping in mind. First, our data was approximate and aggregated, and we did not have access to detailed and accurate data to validate our model. In an ideal world, if we had precise population, location and distance data along with information on the current outreach program, we could estimate the coverage levels predicted by our models for various assumptions on travel preferences and compare these against current coverage levels. We could then use our model to determine the optimal outreach locations for the travel behavior that best captures current coverage and determine the potential improvement.

Second, our results apply mainly to rural outreach settings with relatively lower population densities; in densely populated urban settings coverage models could clearly be much more complex. However, since most urban centers tend to have health posts or clinics with regular hours, outreach generally *is* focused on rural locations.

Third, we assume that the social planner is not biased in favor of outreach plans where the travel is shorter or across easier terrain (which is sometimes the case in practice), and that the plans from our model can be implemented in an unbiased fashion.

Fourth, it is in general, difficult to predict the exact type of coverage applicable to a particular application environment. One study that tried to estimate the willingness of patients in the rural tropics to seek medical care at a primary healthcare facility is described in a paper by Müller et al.  [17], who based their estimates on a database of attendees at a rural clinic in Papua New Guinea. They showed a nonlinear decay in attendance as distance increased but there was considerable heterogeneity depending on age, gender and ailment, and it was not possible to come up with precise numerical estimates. In a more recent paper that is tangentially related, Smith and Harper  [18] describe a possible approach (using Monte Carlo simulation) to track the spatio-temporal spread of the usage of services at a community health center in northern India. Given the general difficulty in accurately estimating coverage levels, we suggest running our models under different assumptions of coverage. As the results indicate, in many instances the optimal locations are identical (e.g., with N=6 locations), with only the estimates of the populations served being different. In other cases there may be some common locations and some that differ (e.g., with N=3), in which case the social planner would make a subjective decision on the locations to select. If it is not clear which of models 1, 2, and 3 is the most appropriate to use, a robustness approach can be adopted. In this case, uncertainty in the coverage assumptions can be handled by looking at how the optimal solution to one model performs when the coverage assumptions correspond to each of the other two models. This can then be used to determine which of the three models is the most robust.

Finally, an important question that our model might raise is the one of equity: since our objective is to maximize the number of children immunized, could it lead to extremely remote villages with low populations being dropped off altogether from coverage? There are different ways to address this issue. One approach that was taken by Smith, Harper and Potts  [19], might be to develop a model with explicit bicriteria efficiency/equity objectives. We opted for a simpler approach. Although our model is static, we envision that it would be re-run with different sets of candidate locations over time, with the idea that over some interval of time every location does get covered. For example, there might be a new outreach cycle each month and over a (say) six month period we could ensure that every location does get covered at least once. One possible drawback with this approach is that a village might not be assigned to the same outreach location each time and this might be troublesome to its residents. An alternative is to perhaps incorporate an equity constraint explicitly into our model. For example in Model 1, we could index each outreach cycle by t=1,2,…,T, and redefine our main decision variables as

$x_{it}\in \left\{0,1\right\}$; 1 if village i is selected as an outreach center in cycle t; 0 otherwise
$y_{it}\in \left\{0,1\right\}$; 1 if village i is covered in cycle t; 0 otherwise.

The constraints could then be readily modified to reflect the requirement that over T cycles every village should be covered. Similar extensions are possible for the other models as well.

In summary, outreach is a critical component of EPI vaccination programs in low and middle income countries. However, there are no standard guidelines for outreach and these activities tend to be conducted in a fairly *ad hoc* fashion. In particular, the problem modeled in this paper is motivated by vaccination activities in India, and our approach is based on adapting facility location models to the outreach coverage problem. Based on past and ongoing work related to vaccine logistics that the authors have done with a number of countries in Asia and sub-Saharan Africa, we feel that these models can aid decision makers when they are establishing outreach policies. The resulting outreach plan affects the performance of the entire vaccine supply chain because the demand for vaccines at all levels of the supply chain will vary with the outreach plan and the resulting vaccine coverage.

## Figures and Tables

**Table 1:** Location information.

Village	Location		Population	Village	Location		Population
	x	y			x	y	
1	−11.84	4.93	228	40	1.53	10.24	401
2	−11.03	−7.51	646	41	1.84	5.19	706
3	−10.07	2.85	366	42	1.94	4.89	650
4	−10.07	3.61	671	43	1.97	−5.86	865
5	−9.88	0.40	594	44	2.76	−9.79	624
6	−9.78	−3.48	624	45	3.18	5.46	273
7	−8.93	3.79	711	46	3.39	−9.66	618
8	−8.88	2.25	475	47	3.43	−4.51	748
9	−7.95	−3.40	198	48	3.96	−7.97	756
10	−7.75	−6.21	561	49	4.59	−7.89	348
11	−7.52	0.19	525	50	4.65	4.82	240
12	−7.13	6.73	1049	51	4.69	2.13	463
13	−6.25	−2.92	554	52	5.08	2.93	434
14	−6.07	0.06	496	53	5.29	−2.83	413
15	−5.37	−1.48	701	54	5.59	9.87	848
16	−5.19	3.30	293	55	5.61	−0.88	584
17	−5.14	−5.06	955	56	5.76	−5.74	661
18	−4.67	−8.98	466	57	6.00	−0.57	636
19	−4.65	11.30	246	58	6.60	−0.74	682
20	−4.54	8.41	203	59	6.71	−8.85	646
21	−4.38	2.98	297	60	6.72	5.99	485
22	−4.14	−3.25	398	61	6.78	−7.45	541
23	−3.60	9.14	254	62	7.12	1.47	792
24	−2.62	−9.70	317	63	7.32	0.80	592
25	−2.38	−6.37	281	64	7.47	8.17	573
26	−1.74	−8.48	736	65	7.48	6.37	423
27	−1.65	−7.33	566	66	7.70	5.60	493
28	−0.89	−10.30	195	67	7.73	1.48	694
29	−0.62	11.76	553	68	7.82	−7.71	470
30	−0.41	−7.66	272	69	8.05	−6.30	482
31	−0.14	8.26	627	70	8.14	−1.24	355
32	0.08	−10.23	543	71	8.90	3.83	692
33	0.24	−8.79	473	72	8.94	3.94	677
34	0.31	−9.84	329	73	9.03	0.51	540
35	0.41	−7.65	374	74	9.53	3.62	90
36	0.62	−10.20	491	75	10.03	5.38	613
37	1.11	6.30	392	76	10.27	6.24	313
38	1.38	−9.63	525	77	12.16	−4.61	488
39	1.49	5.38	348	78	12.88	1.33	456

**Table 2:** Results for the first three models.

N	Model 1: Binary coverage			Model 2: Variable single coverage			Model 3: Variable multiple coverage		
	No. covered	Percent covered	Center locations	No. covered	Percent covered	Center locations	No. covered	Percent covered	Center locations
1	10,749	26.9%	51	14,238	35.7%	53	14,238	35.7%	53
2	20,515	51.4%	**48**, 51	25,167	63.1%	**30**, **51**	25,463	63.8%	**30**, **51**
3	27,417	68.7%	**11**, 48, 51	32,390	81.2%	**11**, **48**, **52**	33,093	83.0%	**11**, **43**, **60**
4	32,257	80.9%	**8**, **17**, 48, 51	35,331	88.6%	**7**, **17**, 48, 52	36,119	90.5%	**5**, **31**, **35**, **62**
5	35,812	89.8%	8, 17, **31**, 48, **62**	37,853	94.9%	8, 17, **31**, 48, **62**	38,347	96.1%	**8**, **17**, 31, **48**, **62**
6	37,590	94.2%	8, 17, 31, 48, **60**, **73**	38,742	97.1%	8, 17, 31, 48, **60**, **73**	39,132	98.1%	8, 17, 31, 48, **60**, **73**
7	39,259	98.4%	**6**, 8, **30**, 31, 60, **69**,**73**	39,572	99.2%	**6**, 8, **30**, 31, 60, **69**, 73	39,746	99.6%	**6**, 8, **30**, 31, 60, **69**, 73
8	39,666	99.4%	**10**, **11**, **23**, **35**, **39**, 60, 69, 73	39,780	99.7%	**10**, **11**, **23**, **35**, **39**, 60, 69, 73	39,844	99.9%	6, 8, **23**, 30, **39**, 60, 69, 73
9	39,894	100.0%	**8**, 10, **22**, 23, 35,39, 60, 69, 73	39,894	100.0%	**8**, 10, **22**, 23, 35,39, 60, 69, 73	39,895	100.0%	**8**, 10, **22**, 23, 35,39, 60, 69, 73

**Table 3:** Coverage at each of 6 centers with different coverage models.

Model	Location no.						Total
	8	17	31	48	60	73	
Binary	5704	6040	3592	9766	6416	6073	37,590
Variable single	5704	6490	3714	9997	6416	6420	38,742
Variable multiple	5971	6425	3665	10,214	6526	6329	39,132

**Table 4:** Coverage with 4 IHCs.

Outreach per IHC	Population	Coverage percentage
1	1387	29.9
2	2243	48.3
3	2810	60.5
4	3169	68.2
5	3416	73.5
6	3607	77.7
7	3743	80.6
8	3816	82.2
9	3846	82.8
